# Sports audiology: Ear hygiene practices of gym users who wear earphones

**DOI:** 10.4102/sajcd.v68i1.794

**Published:** 2021-07-28

**Authors:** Aimee Flowers, Dhanashree Pillay

**Affiliations:** 1 Department of Speech Pathology and Audiology, University of the Witwatersrand, Johannesburg, South Africa

**Keywords:** exercise, ear hygiene, noise induced hearing loss, gym, earphones

## Abstract

**Background:**

Technological advancements have facilitated the demand for electronic systems to track and measure progress during exercise activities. Electronic devices with music applications, such as iPods and smartphones, are popular during gym sessions as they support the ambiance to one’s exercise programme. Earphones are the popular choice for the transmission of music to the gym user’s ear. There is a direct correlation between the cardiovascular health and the aural health because of the effect of increased blood supply to the auditory system. Therefore, this research study explores the ear hygiene of gym users.

**Objectives:**

This study aimed at determining the ear hygiene and middle-ear status of gym users who wore earphones whilst exercising.

**Method:**

A purposive sampling method was adopted. Data were collected in two phases: phase 1 via a questionnaire and phase 2 included an otoscopic examination and tympanometry. Qualitative and quantitative data were analysed.

**Results:**

Fifty-four participants were included. Ear hygiene was not a priority for all gym users. The results revealed that 87% of the participants randomly cleaned their ears; however, 59% were cleaning their earphones. The middle ear assessment indicated that 17% of the participants presented with pathological indicators. Participants requested for the implementation of educational programmes pertaining to infection control measures for proper ear hygiene.

**Conclusion:**

There is a dearth of health and wellness educational programmes that include ear hygiene practices, necessitating the development of prevention and promotion programmes for this niche area in audiology. The need for guidelines pertaining to ear hygiene practices amongst gym users is vital to ensure that the auditory system is monitored and protected when individuals participate in exercise sessions.

## Introduction

The 19th century saw the first industrial revolution in the textile industry as new production methods were employed (Crafts, 2011; De Pleijt, Nuvolari, & Weisdorf, 2020; Mohajan, 2019). The first, second and third industrial revolutions have created the foundation for the technological developments in the dynamic fourth industrial revolution in the 21st century. The fourth industrial revolution has begotten a human lifestyle that centres on the use of technologically advanced systems and devices in almost every area of life (Schwab, 2017; Xu, David, & Kim, 2018).

The World Economic Forum (2020) states that:

The 4th Industrial Revolution is about more than just technology-driven change; it is an opportunity to help everyone, including leaders, policy-makers and people from all income groups and nations, to harness converging technologies in order to create an inclusive, human-centred future.

Economic, educational, social and political spheres depend on the technology to communicate, learn and live. Individuals use technology connected to earpieces such as earphones and insert ear devices to socialise, to assist in health and wellness and to gain new knowledge. The World Health Organization describes good ear hygiene as follows (WHO, 2006:1):

- not using medication in the ear that is not prescribed for you
- not using dirty towels to clean one’s ears
- not putting anything into your ears
- not cleaning one’s ears with hairpins, toothpicks or anything else
- not putting water into one’s ears
- not leaving cotton wool in one’s ears unless directed by a healthcare worker.

Individuals who wear ear devices are advised to follow good ear hygiene practices, such as cleaning and disinfecting the ear devices regularly (Gompa & Anand, 2019; Mazlan, Thomas, Saim, & Liyab, 2002).

Focal areas in the field of Audiology in the 21st century depend on the use of electronic ear devices for the assessment and management of hearing loss and ear care. Auditory brainstem assessment, otoacoustic emission assessments and personal amplification systems are but a few technological advancements in the current century that has provided the opportunity to assess and manage auditory-related disorders in an effective, efficient and precise manner (Iliadou et al., 2017). Despite the electronic advancements for assessment and diagnosis of an auditory pathology, human-centredness and prevention are still at the forefront of care in Audiology.

Ear-related electronic devices are popular in the 21st century, necessitating the promotion of ear care and the prevention of auditory pathologies. Earphones are the most commonly used electronic ear device amongst the general population as individuals use earphones for various activities, such as listening to music, podcasts and audiobooks, watching movies, gaming, and making phone calls (Hiipakka, 2015). Earphones are most commonly used by people for enjoying music, especially during exercise sessions (Barney, Gust, & Liguori, 2012; Jones & Ekkekakis, 2019). The earphones placed at the entrance of the ear canal poses a risk for developing otitis media (OM), otitis externa (OE) and a noise-induced hearing loss (NIHL) as the earphones are carriers of dust and microbial particles and they may emit excessive noise (Mazlan et al., 2002).

International studies identified that gym users are at a greater risk as exercise exacerbates the physiological changes and increases the ear’s susceptibility to auditory pathologies, such as OM and OE (Vittitow, Windmill, Yates, & Cunningham, 1994). Good ear hygiene practices are not limited to protecting one’s hearing, but it includes cleaning ear accessories, such as earphones (Chadha, 2014; WHO, 2012). However, there is a dearth of research pertaining to the ear hygiene practices of the gym users who wear earphones.

Audiologists in South Africa (SA) play an important role in the promotion of good ear hygiene practices and prevention of auditory pathologies. Globally, the research study on sports audiology focuses on social and professional sports participants (Du Plessis, Fothergill, Gertner, Hughes, & Schwaller, 2008; Inalsingh, 2012; Pillay & Dada, 2018; Pillay & Jardine, 2010); however, there is a lack of studies that focus on the ear hygiene practices of gym users. Gym users may not be considered as sportsmen or women; however, gym users participate in training akin to sports people. Therefore, a study pertaining to the ear hygiene practices and earphone usage amongst gym users would provide valuable evidence to support additional investigations in the area of sports audiology.

## Background

Playing and watching sports are popular recreational activities globally. Increased participation in sports can be attested to social connects that are created during the shared participation and enjoyment of the sport (Department of Sports and Recreation, 2005). South Africa has an emphasised interest in sports (Department of Sports & Recreation, 2005), as South Africans are those who take their sports seriously (eds. Alegi & Bolsmann, 2013).

Sports provide escapism and a healthy competitive atmosphere in SA (Department of Sports and Recreation, 2005) as people are becoming more aware of the physical and mental well-being. Exercise and physical training were synonymous with sportsmen and women; however, wellness and fitness are growing in their popularity with the general population. Globally, individuals have become more attuned to a healthier lifestyle, and gyms have become spaces that are frequented by the average person on a daily basis.

It is estimated that, globally, there are 58 000 000 individuals who utilise gyms, on average, two times per week (Statistic Brain, 2017). The number of people attending and exercising at gyms has also increased in SA (Fitness and Health, 2017) despite the insufficient statistics on gym attendance in SA. South African gyms earn R12.5 billion a year, which is the highest in the world (Mapumulo, 2017), suggesting that a significant number of South Africans attend gym on a regular basis.

Health professionals promote exercise to increase immunity and reduce stress levels (Gleeson, 2005) as exercise releases endorphins. Exercising demonstrates a positive impact on health; however, confounding variables whilst exercising can have negative impact on the health of the individual. Gyms are designated spaces for exercise, which focus on a healthy physical lifestyle; however, there are situations and activities within the gym that have the potential to harm the gym user.

Exercise and training at a gym may have a negative impact on the individual because of an increase in blood pressure levels, thus increasing pressure to the auditory system and brain (Delp et al., 2001), as well as increasing the risk of middle ear infections and hearing loss because of earphone usage. In addition to the straining, individuals may hold their breath to assist them when lifting the weights, resulting in an increased pressure within the auditory system. Intense yoga poses, the loud noise level in group sessions and noise from crashing weights are incidental factors that can negatively impact the auditory system. Abnormal pressure in the auditory system related to participation in sports and exercise may lead to the formation of a perilymphatic fistula, tinnitus, dizziness and hearing loss (Assi, Moore, Ellemberg, & Hébert, 2018; Vavilala et al., 2018; Vogel, 2014). Gyms provide the user with the opportunity of participating in classes, such as boxing and swimming sessions (Inalsingh, 2012), which could impact the auditory system if ear hygiene practices are ignored, thus, resulting in a possibility of increased ear infections and hearing loss.

Whilst there is a risk of harmful side-effects of exercise, it is evident that a healthy lifestyle requires exercise. Wellness programmes reinforce the positive effects of exercise on the overall physical and mental aspects of human life. The technological advances of the fourth industrial revolution have brought with it health and wellness accessories and wearables, such as wristbands, watches and hearables. Hearables are wireless in-ear computational devices that fit inside an individual’s ear canal and enhance the quality of listening (Banks, 2015). Hearables are supplemented with biometric personal identification information, vital sign tracking, activity tracking, augmented hearing and improved sound quality through the filtering of sound and a decrease in background sound interferences (Banks, 2015). The design of hearables must ensure that a seal is created within the ear canal to limit sounds from escaping. The concern arises when high-intensity music is played through the hearables as reverberation increases with hearables when compared with a free-field environment (Dobie & Van Hemel, 2005). Determination of safe listening levels is based on free-field listening and not hearables, such as earphones (Dobie & Van Hemel, 2005), highlighting the need for further research.

A research study conducted on the effects of earphones focuses on call centre agents (Trompette & Chatillon, 2012) and young adults who use personal listening devices (Sulaiman, Husain, & Seluakumaran, 2015). A healthy auditory system is linked to good ear hygiene practices, such as cleaning one’s ears, cleaning the devices used in one’s ears and decreasing noise exposure (American Academy of Otolaryngology–Head and Neck Surgery, 2017; Chadha, 2014; WHO, 2012). There is a dearth of research pertaining to ear hygiene practices. The studies conducted revealed that the majority of individuals are unaware of or are misinformed about the correct ear hygiene practices, and those with some knowledge choose to ignore the recommendations as they believe the harmful effects do not apply to them (Punch, Elfenbein, & James, 2011). The misperception that ear health refers to the removal of excess wax using earbuds (Olaosun, 2014) can result in ear drum perforations and hearing loss (Prusick, 2015). Ear hygiene includes the cleaning of the pinna and the devices that are inserted into the ear canal, which is a common practice during a hearing assessment conducted by an audiologist as infection control is mandatory (Bankaitis, 2015). Audiologists have the knowledge to assist gym users in proper ear care and infection control practices, and hence, their role with gym users.

The rationale for this study stemmed from the lack of specific educational infection control programmes available in SA targeting individuals who wear hearables on a social basis, despite the increased use of hearables during exercise programmes and at events, such as silent discos and secret sunrise exercise gatherings (Secret Sunrise, 2018; Silent Events, 2018). The lack of awareness programmes to support good ear health will lead to the lack of knowledge pertaining to ear hygiene practices, resulting in a possible diagnosis of OM or OE (Waitzman, 2017). The earliest study in 1985 illustrated the positive link between OM and the use of airline headsets (Brook, 1985). Otitis media is directly caused by Staphylococcus bacteria, and more recent studies have supported the evidence that unclean earphones carry these bacteria (Mukhopadhyay, Basak, Gupta, Chawla, & Bairy, 2008). Gym users who wear hearables are at a higher risk as their sweat from exercise creates a breeding ground for bacteria, as it creates a moist and warm environment that is ideal for Staphylococcus to grow (Bhatia & Zahoor, 2007).

Individuals who were exposed to noise during exercise performed significantly worse when assessed for temporary threshold shifts when compared with those who were exposed to noise only and exercise only (Lindgren & Axelsson, 1988; Vittitow et al., 1994). Exercise causes an increase in heart rate and blood pressure, which may, in turn, affect the blood circulation to the cochlea (Vittitow et al., 1994), and by wearing unclean earphones and listening to music at extreme intensities, the auditory system is at a greater risk for developing OM and/or a hearing loss. Therefore, it is crucial to document and profile the ear hygiene practices of gym users who wear earphones.

The current study aimed at contributing to new knowledge within the area of sports audiology in SA, and it plays an integral role in building foundational information pertaining to ear hygiene practices of gym users.

## Research method and design

### Aims of the study

- To document any significant audiological case history information.
- To document the ear hygiene practices and earphone usage of gym users.
- To assess and document the tympanometry results of gym users who wear earphones.

### Research design and instrumentation

This study employed an exploratory, mixed method research design. The data were collected in two phases: phase 1 utilised a questionnaire that was developed by the researcher to obtain qualitative data, and phase 2 included an otoscopic examination and tympanometry assessment that yielded quantitative data.

### Sampling and sample

A purposive sampling method was undertaken to identify individuals who attended gyms. The sample comprised of individuals who were familiar with the researcher and individuals who were recruited via an advert on social media.

Fifty-four participants between the ages of 18 and 50 years were included in this study, 26 male and 28 female. Participants met the following inclusion criteria: participants were required to attend and exercise at a gym for 3 days a week or more; they were required to own a pair of earphones, which they wear during the exercise sessions; they needed to be over the age of 18 years and could identify with any gender description or nationality.

The data were collected at a location that was convenient for the participants and were then documented on a record sheet. The otoscopic examinations were performed by the researcher to ensure consistency and reliability of results. The researcher documented all findings onto the record sheet. The findings, such as impacted wax, abnormalities of the pinna and ear canal, were recorded. The middle-ear status was assessed via a calibrated tympanometer with a 226-Hz probe tone, which is an appropriate choice for the adult participants in this study. The tympanogram classification was based on the Jerger (1970) classification system for tympanometry, which utilises types of tympanograms as per the normative values.

Participants received the results of the middle ear assessment immediately, and were counselled and referred to the appropriate healthcare practitioners if needed.

### Ethical considerations

Ethical clearance was granted by the University of the Witwatersrand Medical Research Ethics Committee in 2018 (clearance number: M180216), and data were collected thereafter. Potential participants were provided with an information sheet, detailing the research methods and requirements. Individuals who agreed to participate were provided with an informed consent form. Descriptive statistics were employed for the analysis of the quantitative data.

## Results

The results are presented as per the aims of the study.

### Aim 1: To document any significant audiological case history information

Five participants experienced multiple ear infections during childhood, of which two had undergone surgery to insert grommets and the other three had a history of ear infections as a result of swimming. Seven participants were experiencing ear problems at the time of data collection, which included an excessive wax build-up, tinnitus and hearing loss. Eight participants experienced dizziness after exercising, and 11 participants experienced tinnitus. Thirteen participants experienced a change in hearing ability after strenuous training sessions whilst listening to loud music via earphones. Participants revealed that the loss of hearing lasted for 30 sec to an hour.

### Aim 2: To document the ear hygiene practices and earphone usage of gym users

Forty-seven participants indicated that ear cleaning formed a part of their hygiene practices. The number of times that participants cleaned their ears varied from every day to every second month. Twenty participants practised daily ear cleaning, 20 of them practised weekly ear cleaning, three practised monthly ear cleaning and four participants cleaned their ears at an interval of two or more months. Ear cleaning methods and tools were identified as earbuds (29), water (10), soap and water (8), cloth (6), finger (4), tissue (1), waxsol (1), syringe (1) and bobby pin (1).

Participants’ earphone hygiene practices revealed that 32 participants cleaned their earphones; however, there were variations in the frequency of cleaning practices. Three participants practised daily earphone cleaning, 15 practised weekly cleaning, six practised monthly cleaning and seven cleaned their earphones every few months when it became visibly dirty. Methods of earphone cleaning varied between using a cloth (9) to using chemicals, such as Dettol wipes (1), alcohol swabs (2), chlorohexidine (1), germicide (1), hand sanitizer (1), cologne (1) and surgical spirits (1). One of the participants used a safety pin to clean the earphones.

Forty-six participants indicated their willingness to participate in education programmes geared at ear hygiene practices. Participant 4 stated that ‘the implementation of community programmes at the level of primary intervention would prevent future complications’. Participants indicated that their understanding and perceptions surrounding ear hygiene were limited and poor.

The average participant exercised three or four times a week and utilised earphones at every session. The mean volume level used by the participants to hearing music was at 70% of the maximum volume on the device.

### Aim 3: To assess and document the tympanometry results of gym users who wear earphones

The results are presented in terms of ears rather than participants. The otoscopic examination revealed that 90 ears were free from abnormalities, five ears presented with a partial wax occlusion, 11 ears were fully occluded with wax and two ears showed visible exostoses. The participant who presented with the exostoses indicated that he or she cycles outdoors on a regular basis.

The tympanometry results revealed that 90 ears presented with Type A tympanograms, two ears with Type As tympanograms, two ears presented with Type B tympanograms and three ears with Type C tympanograms.

## Discussion

### Middle-ear factors

Eleven per cent of participants presented with a full wax occlusion and 4% with a partial wax occlusion. This number is quite large in relation to the sample size. The presence of wax is normal and healthy; however, it becomes problematic when it blocks the ear canal as it may decrease hearing thresholds (Prusick, 2015). The use of earphones may result in a wax build-up and ear canal occlusion (Beaudoin, Davies, Lee, & Zalisk, 2020). The function of wax is to protect the ear from dust, harmful bacteria and infection. A research study shows that earphones are vectors for bacteria, and by placing them in the ear, the risk of infection increases (Schwaab, Gurr, Neumann, Dazert, & Minovi, 2011). The findings from the current study suggest a relationship between the use of earphones, occluded ear canals and a hearing loss. An explanation for the increased wax production may be a protective mechanism to reduce the risk of ear infection (Schwaab et al., 2011). A study conducted in 2002 by Mazlan et al. exploring the relationship between ear infections and earphone users found that four participants presented with a wax impaction. Therefore, the researcher concluded that it could not be attributed to the earphones. Therefore, a larger, more detailed study would provide valuable evidence of the significant relationship between the three variables. Additionally, the participant who presented with the exostoses indicated that he or she cycles early in the morning and often encounters cold conditions. The weather conditions play a role in the possible development of exostoses (Mazza, 2016), which highlights a possible relationship between the presence of exostoses in the ear canals of individuals who cycles under the cold morning conditions. Exostoses are benign bony growth that form in the outer ear canal, most commonly after repeated exposure to cold water (Moore et al., 2010). However, exostoses can occur after a repeated exposure to extremely cold air (Mazza, 2016). Therefore, audiologists must be cognisant of the effects of weather conditions on sportswomen and men.

Type B and C tympanograms can be related to middle-ear pathologies, such as OM (Onusko, 2004; Passali et al., 2014). Otitis media and OE are reported in individuals who swim (Jayakar, Sanders, & Jones, 2014; Wiegand, Berner, Schneider, Lundershausen, & Dietz, 2019; Wipperman, 2014), and as a result, there is a need for grommets to manage the middle-ear infections (Stephen, Leach, & Morris, 2013). The current study found that two gym users utilised the pool facilities to exercise, and therefore, individuals who swim and have pre-existing ear pathologies should receive advice on ear care to protect the middle-ear system from further damage (Wang, Liu, Shiao, & Wang, 2005). The use of earphones during exercise sessions exposes the user to bacteria from the earphones (Liaqat, Tariq, Jamil, & Amin, 2015) and participants who have a hearing loss and who experience tinnitus are innately susceptible to further ear damage. A research study suggests that bacteria live on earphones in the form of biofilm, thus, creating a vector for causing ear infections (Liaqat et al., 2015).

### Noise levels

Participants who revealed that they have a hearing loss did not disclose the cause of the hearing loss; however, it is vital to monitor the noise levels when using earphones during exercise sessions in order to prevent further damage on the hearing mechanisms. The noise exposure in gyms may be because of loud music during group classes, weights crashing and general music noise within the gym. The current study highlights the need for a large-scale study that monitors the noise levels in gyms as exercising results in the auditory system being more sensitive to sound (Vittitow et al., 1994). The experience of tinnitus and hearing loss after training may be attributed to loud music levels (Ireland, 2017), and the results from participants in the current study revealed that music volume levels were 70% of the maximum volume. The temporary shift in thresholds after exposure to loud music is common (Le Prell et al., 2012; Zhao, Manchaiah, French, & Price, 2010), which highlights the need for monitoring the hearing status of individuals who are exposed to loud music during gym sessions.

A study by Punch et al. (2011) suggested that the participants were aware of the dangers of listening to music at loud levels; however, they underestimate the damage of noise that was caused to the auditory system. Educational programmes are suggested to deliver preventative measures in a more effective, long-lasting manner (Punch et al., 2011).

### Intense exercise

Intense workout sessions increase the susceptibility of developing audiological symptoms (Ireland, 2017) as strenuous workouts, such as cardiovascular exercise, resistance training, strength and stretch training, and high-intensity workouts, increase the blood flow to the cochlea, resulting in an increased sensitivity whilst training (Welch, Law, & Dirks, 2014). The negative effects of intense workout sessions include increased middle-ear pressure that results in a possible perilymphatic fistula (Gurgel, 2014; Magliulo, Stasolla, Colicchio, & Gagliardi, 2010) The current study revealed that participants are experiencing audiological symptoms after training, and these symptoms may be warning signs of the hazardous effects of exercise on the auditory system. The negative effects of exercise on the auditory system may also be compounded because of the increase in middle-ear pressure (Nall, 2017). Therefore, education on the effects of illness on the auditory system and the compounding effect of exercise on a compromised auditory system is important.

### Ear hygiene

The ear is a self-cleaning mechanism, and it is, therefore, recommended that individuals do not need to clean their ears (Prusick, 2015). However, individuals from the current study described various cleaning methods, some of which are harmful. These included the use of earbuds and a bobby pin. Individuals are generally misinformed or are not aware of the correct ear hygiene practices (Olaosun, 2014). The results of the current study correlated with those of a study exploring the ear cleaning habits amongst educated young adults in Nigeria (Olaosun, 2014). The current study and the study conducted by Olaosun (2014) found that individuals are unaware of the correct manner in which to clean the ear. Participant 2 stated that they felt that ‘there is not enough awareness surrounding aural hygiene’. Therefore, it is important to educate the gym users on how to care for their ears and avoid further damage from using earphones.

Education provides individuals with the tools they need to identify problems and create solutions (Zimmerman, Woolf, & Haley, 2015). Educating the public about healthcare is extremely important in order to promote hygiene practices and prevent diseases. The current study revealed that there is a need to educate individuals about the status of their middle-ear health whilst exercising. The warning signs and symptoms of various audiological disorders should be displayed in gym sites to create awareness. It is evident that health and wellness programmes need to consider the inclusion of auditory assessments in order to ensure that aural health and communication are monitored.

## Conclusion

Exercise is vital in the overall healthy lifestyle regime; therefore, one should be equipped with the knowledge of preventing the audiological abnormalities that could result during exercise sessions. Various types of exercise activities have different effects on the audiological system and may cause audiological symptoms during and after training sessions. By providing the gym user with the required ear hygiene knowledge, the audiologist can promote good ear health and prevent ear damage. There is a need for aural hygiene awareness, especially in the gym setting, and the role of the audiologist is highlighted in this niche area. The audiologist can consider the following recommendations:

- Lobby that medical aids create a mandatory hearing assessment within their health and vitality programmes, thus, promoting the monitoring of one’s hearing status.
- Make requests to the gyms for posters that indicate the need to disinfectant surfaces, including one’s earphones.
- Offer screening at gym sites to promote awareness of aural hygiene and hearing.
- Petition the producers of hearables to request for a warning sign to be placed on hearables to prevent misuse at loud volumes.
- Petition the producers of earphones and request that cleaning instructions are made available within the instruction manual.

Exercise is necessary for a healthy body and mind; therefore, one cannot stop exercising in order to decrease the risks of auditory damage. However, audiologists play a significant role in educating and equipping the gym users about ear hygiene and care. There is a niche area of sports audiology, which should be explored in a deeper manner.
